# Targeted metabolomic analyses of cellular models of pelizaeus-merzbacher disease reveal plasmalogen and myo-inositol solute carrier dysfunction

**DOI:** 10.1186/1476-511X-10-102

**Published:** 2011-06-17

**Authors:** Paul L Wood, Tara Smith, Lindsay Pelzer, Dayan B Goodenowe

**Affiliations:** 1 Phenomenome Discoveries Inc, 204-407 Downey Road, Saskatoon, SK S7N 4L8, Canada

**Keywords:** leukodystrophy, Pelizaeus-Merzbacher disease, fibroblasts, lymphocytes, 158JP oligodendrocytes, plasmalogens, myo-inositol transporter, peroxisomal disorders

## Abstract

**Background:**

Leukodystrophies are devastating diseases characterized by dys- and hypo-myelination. While there are a number of histological and imaging studies of these disorders, there are limited biochemical data available. We undertook targeted lipidomic analyses of Pelizaeus-Merzbacher disease (PMD) fibroblasts, PMD lymphocytes, and 158JP oligodendrocytes, a murine model of PMD, to define the lipid changes in these cell models. Further targeted metabolomics analyses were conducted to obtain a preliminary evaluation of the metabolic consequences of lipid changes and gene mutations in these cell models.

**Results:**

In both PMD fibroblasts and lymphocytes, and 158JP oligodendrocytes, ethanolamine plasmalogens were significantly decreased. Labeling studies with 158JP oligodendrocytes further demonstrated a decreased rate of lipid remodeling at sn-2. Targeted metabolomics analyses of these cells revealed dramatic increases in cellular levels of myo-inositol. Further uptake studies demonstrated increased rates of myo-inositol uptake by PMD lymphocytes.

**Conclusions:**

Our data demonstrating PlsEtn decrements, support previous studies indicating leukodystrophy cells possess significant peroxisomal deficits. Our data for the first time also demonstrate that decrements in peroxisomal function coupled with the PLP1 gene defects of PMD, result in changes in the function of membrane myo-inositol solute carriers resulting in dramatic increases in cellular myo-inositol levels.

## Background

The leukodystrophies include a heterogeneous group of both childhood and late onset genetic diseases that primarily result in dys- or hypo-myelination [1,2]. Furthermore, these disorders are highly misdiagnosed such that disease incidence is much greater than previously thought [3]. Neuroimaging has significantly improved the ability to detect the CNS deficits in these disorders. However, there is limited biochemical knowledge of the underlying disease processes. Therefore, we undertook a targeted lipidomics analysis of the established peroxisomal deficits in PMD fibroblasts [4,5], PMD lymphocytes, and 158 JP oligodendrocytes [6], all of which demonstrate a proteolipid protein-1 (PLP1) mutation. A targeted metabolomics analysis of the consequences of PLP1 mutations on cellular metabolism also was conducted.

## Materials and Methods

### Cell Culture

The following cell lines were analyzed: two murine oligodendrocytes cell lines, 158N (normal) and the PLP1 mutant 158JP (Jimpy) (a generous gift from Dr. S Ghandour); control human lymphocytes (Coriell GM00131 and GM02184); human PMD lymphocytes (Coriell GM09545); human fibroblast controls (Coriell GM00409 and ATCC CRL-2076) and human PMD fibroblasts (Coriell GM09546). All fibroblast cell lines and oligodendrocytes were cultured (10 cm^2 ^plates) in DMEM:F12 (Mediatech) supplemented with 15% FBS (Invitrogen) and 1% antibiotic/antimycotic (Invitrogen). Lymphocyte cell lines were suspension cultures (25 ml flasks) in RPMI 1640 (Hyclone) supplemented with 10% FBS and 1% antibiotic/antimycotic. All cells were grown at 37°C in a 5% CO_2 _incubator. Fibroblast cells and oligodendrocytes were harvested when plates reached confluence using a cocktail of Versene and TryPLe express (2:1; Gibco). For all cells the pellet (1280 xg) was washed twice with phosphate buffered saline (PBS) and the stored at -80°C for subsequent analyses.

### RNA Isolation and Quantitative Real-Time PCR

Total RNA was isolated from confluent T-25 flasks of GM00131, GM02184 and GM09545 using the RNeasy Mini Kit (Qiagen) as per the manufacture's protocol (n = 4). Quantification of RNA was performed by optical density with the NanoVue spectrophotometer (GE Healthcare Life Sciences). Reverse transcription reactions were performed on 1 μg RNA using the qScript cDNA SuperMix (Quanta Biosciences). Each sample was analyzed to determine expression of the housekeeping gene β-actin (sense-5' agccatgtacgtagccatcc 3'; antisense-5' ctctcagctgtggtggtgaa 3') as well as SMIT1 (sense-5' gctacgagctggctttaatcct 3'; antisense-5' tttactcaggtgctggaggagaa 3') [7] and SMIT2 (sense-5' gcctccacagttagatcccc 3'; antisense-5' cagaactagcaccgcgatgt 3') [8]. Specificity of each primer set was determined by analysis of the dissociation curve. Quantitative real-time PCR was carried out in triplicate using the Fast SYBR Green Master Mix (Applied Biosystems) on the StepOne Plus Real-Time PCR System (Applied Biosystems). Thermocycling conditions were: 95°C for 20s followed by 40 cycles of 95°C for 3s and 60°C for 30s.

### Plasmalogen Synthesis

To monitor plasmalogen synthesis in 158N and 158JP oligodendrocytes, cells were incubated with 100 uM PPI-1038 for 72 hr and incorporation into cellular plasmalogens measured. PPI-1038 is an ether lipid plasmalogen precursor with a [^13^C_3_]glycerol backbone, a [^13^C_16_]palmitic ether linkage at sn-1, a [^13^C_3_]DHA acyl linkage at sn-2, and a lipoic acid acyl linkage at sn-3 to stabilize the precursor.

### Plasmalogen Analyses

For plasmalogen analyses, cells were sonicated in 1 mL of PBS + 0.5 mL methanol. Next, 2 mL tert-butylmethylether were added and the samples capped and shaken (1400 rpm) for 10 min at room temperature. The samples were then centrifuged for 8 min in a clinical centrifuge and 1 ml of the upper organic layer isolated for LC-MS/MS analyses of endogenous and labeled ethanolamine plasmalogens as reported previously [9].

### Myo-Inositol Analyses

Harvested cells were sonicated in 1.2 ml of acetonitrile:MeOH:formic acid (800:200:2.4) containing [^2^H_6_]myo-inositol internal standard. These cell lysates were centrifuged at 4°C and 25,000xg for 30 min. Two 400 μL aliquots of the supernatant were transferred to 1.5 ml screw top microtubes and dried in a Savant centrifugal evaporator. For myo-inositol analysis, timethylsilylation of the samples was conducted at 80°C for 1 hr with 100 μL acetonitrile and 100 μL of N, O-bis(trimethylsilyl)trifluoroacetamide and TMCS (10/1). The TMS derivatives were analyzed by GC-MS with the [MH]^+ ^cations of 613.2 and 619.2 monitored for myo-inositol and [^2^H_6_]myo-inositol, respectively. GC-MS analyses were performed with an Agilent 7890A GC and an Agilent 5975C mass analyzer, with ammonia as the reagent gas. The GC column was a 30 m HP-5MS (0.25 mm ID; 0.25 μm film).

### Myo-Inositol Uptake

Cells were incubated for 0.5, 1, 2, and 3 hours with HBSS-HEPES, containing MEM vitamins and 200 uM [^2^H_6_]myo-inositol. At the end of the incubation cells were washed with ice-cold PBS and processed as described above. Intracellular [^2^H_6_]myo-inositol was monitored as described above using [^13^C_6_]glucose as the internal standard.

### Data Analyses

Data are presented as mean ± SEM for groups of five to six 10 cm^2 ^plates or 25 ml flasks. Since standards are not available for the lipidomics analysis of plasmalogens, these were normalized to the housekeeping metabolite PtdEtn 16:0/18:0. For myo-inositol analyses, concentrations were expressed on a per mg protein basis [10]. GC-MS analyses were performed using 5 point standard curves (reference standards at 0.2 to 10 times the stable isotope internal standard). Data were analyzed by t-test.

## Results

### Ethanolamine Plasmalogens

Peroxisomal deficiency is known to result in plasmalogen deficiency [13].

**Figure 1:**
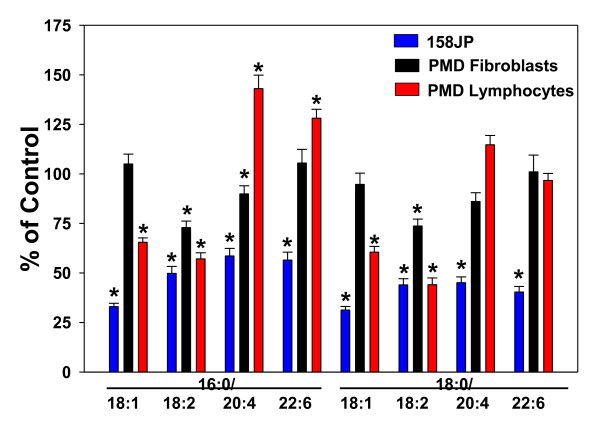
**Ethanolamine plasmalogen levels in 158JP oligodendrocytes and Pelizaeus- Merzbacher disease (PMD) fibroblasts and lymphocytes**. N = 6. *, p < 0.01 vs. control. Mean ± SEM.

In the case of 158JP oligodendrocytes, both white matter ethanolamine plasmalogens (PlsEtn 16:0/18:1; 16:0/18:2, 18:0/18:1; 18:0:18:2) and gray matter PlsEtn(16:0/20:4, 16:0/22:6, 18:0/20:4, 18:0/22:6) were decreased by 50 to 75% (Figure 1). Plasmalogen decrements in PMD fibroblasts were not global, being restricted to plasmalogens with 18:2 or 20:4 at sn-2. These decreases were 10 to 30% (Figure 1). Plasmalogen decrements in PMD lymphocytes also were not global, being restricted to plasmalogens with 18:1 or 18:2 at sn-2. These decreases were 14 to 56% (Figure 1).

### Ethanolamine Plasmalogen Synthesis

**Figure 2:**
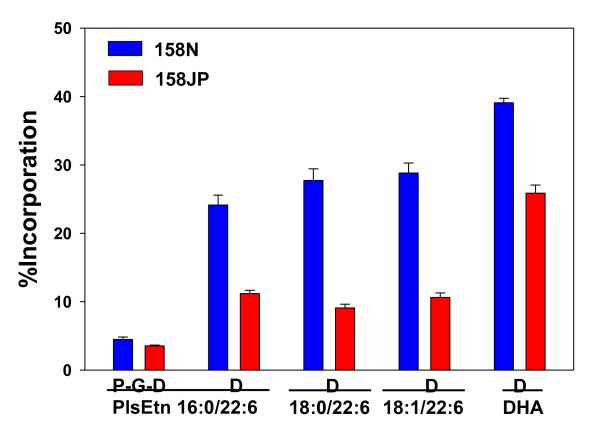
**Incorporation of PPI-1038 (100 μM; 72 hr) into the 16:0/22:6, 18:0/22:6 and 18:1/22:6 plasmalogens of 158N and 158JP oligodendrocytes**. N = 5. P = [^13^C_16_]palmitic acid; G = [^13^C_3_]glycerol; D = [^13^C_3_]DHA.

Incorporation of intact labeled PPI-1038 (i.e. P-G-D = [^13^C_16_]Palmitate-[^13^C_3_]Glycerol-[^13^C_3_]-DHA) into the target plasmalogen (P-G-D PlsEtn 16:0/22:6) was not significantly different between 158 N and 158 JP oligodendrocytes (Figure 2). However, lipid remodeling, namely deacylation/reacylation at sn-2 was significantly decreased (Figure 2).

### Probe Targeted Metabolomics Study

Targeted metabolomics analysis of cells was undertaken utilizing four GC-MS panels that assay over 100 metabolic intermediates in amino acid, nucleotide, alcohol, sugar, polyol, fatty acid, and organic acid pathways. The most dramatic alterations observed in this probe study were increases in cellular myo-inositol levels.

### Myo-Inositol Co-Transporters

**Figure 3:**
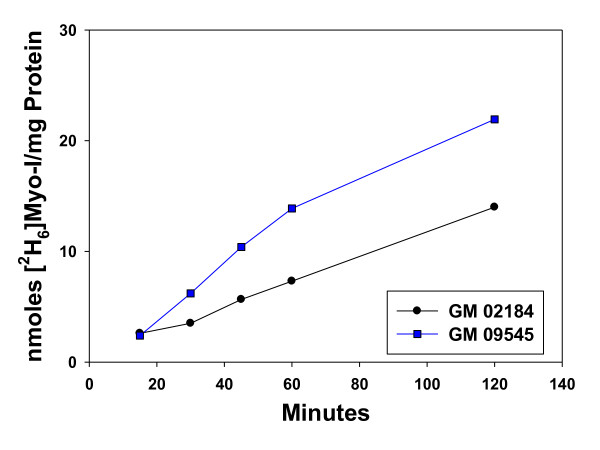
**Uptake of [^2^H_6_]myo-inositol (200 uM) in B184 (control) and PMD45 lymphocytes**. Values are the average of duplicates. The initial rates of uptake were 0.111 ± 0.0039 nmoles/mg protein/min in control lymphocytes and 0.258 ± 0.0062 in PMD lymphocytes.

Myo-inositol levels were significantly elevated in 158JP oligodendrocytes and in PMD fibroblasts and lymphocytes (Table 1), all of which possess PLP mutations. Studies of [^2^H_6_]myo-inositol uptake demonstrated that changes in cellular levels were reflected by 2.3-fold increases in the uptake rate of myo-inositol in PMD lymphocytes (Figure 3). PCR analyses of lymphocytes demonstrated the presence of both SMIT1 and SMIT2 but no difference in expression between PMD and control cells.

**Table 1:** Myo-Inositol levels in 158JP oligodendrocytes, PMD fibroblasts and PMD lymphocytes

Cells	Myo-Inositol (-fold control)
158JP oligodendrocytes	8.7 ± 0.78

PMD Fibroblasts	6.8 ± 0.55

PMD Lymphocytes	1.9 ± 0.10

N = 5 (repeated 3 times); all increases are significant (p < 0.01)

## Discussion

The role(s) of peroxisomal dysfunction in leukodystrophies remains to be clearly defined. Both endoplasmic reticulum (ER) dysfunction (due to accumulation of mis-folded proteins in the ER of PMD cells [11-13]) and peroxisomal dysfunction may be involved in the plasmalogen decrements monitored in this study since both of these cellular compartments are essential for plasmalogen synthesis. Addition of the phosphoethanolamine group at sn-3 [EC 3.1.3.4] of the glycerol backbone and the desaturation of the ether linked fatty acid at sn-1 [EC 1.14.99.19], both occur in the ER [11]. In addition, the many complex interactions between peroxisomes, mitochondria and the ER [14] may be important in the biochemical changes monitored in leukodystrophy cells. This dysfunction is further reflected by the decreased lipid remodeling of plasmalogens via deacylation and reacylation at sn-2. Such decrements in lipid remodeling will also have negative effects on cellular signaling [6].

The decreases in both white matter and gray matter plasmalogens we measured in PMD fibroblasts are consistent with the decreases in white matter [15] and neuronal loss [16] in PMD patients. It is not unexpected that alterations in membrane plasmalogens, which represent 85% of the myelin lipid pool, and in PLP, which represents 50% of the myelin protein pool, might alter membrane transporter function in this complex disease. Prior research has demonstrated that plasmalogen deficiency [17-19] and PLP mutations [20] result in decreased cellular export of cholesterol. Negative effects on other transporters in PMD are to be anticipated as a result of these complex changes in membrane ultrastructure. Previous NMR data have demonstrated increases in PMD brain myo-inositol [15]. Our data in PMD fibroblasts and lymphocytes and in 158JP oligodendrocytes support these findings and demonstrate that increased myo-inositol uptake is responsible for these changes. *In toto*, these data suggest that changes in membrane plasmalogens and/or membrane PLP lead to altered function of sodium/myo-inositol transporters SMIT1 and SMIT2 [7,8,21,22], resulting in cellular accumulation of myo-inositol.

In addition to PMD [15], *in vivo *magnetic resonance spectroscopy studies have demonstrated increases in brain myo-inositol in a number of other leukodystrophies including Krabbe disease [23], infantile Alexander disease [24], metachromatic leukodystrophy [25], adult X-linked adrenoleukodystrophy [26], and childhood X-linked adrenoleukodystrophy [27]. In childhood X-linked adrenoleukodystrophy [27], increases in myo-inositol appear to coincide with the onset of hypomyelination. Myo-inositol accumulation has also been implicated in the etiology of peripheral neuropathies [28]. Our data, combined with the rich publication record of magnetic resonance spectroscopy studies suggest that elevated myo-inositol levels in brain white matter tracts may be a major determinant of hypomyelination. Changes in osmotic pressure resulting from altered myo-inositol transport [29] could represent an initiating event in the cell death of oligodendrocytes as further reflected by the increased osmotic fragility of myelin lamellae in PLP-null mice [30].

In summary, PLP mutations are known to result in dysfunction of the complex organelle interplay of peroxisomes, mitochondria and ER thereby affecting trafficking of critical membrane proteins and phospholipids. While it is well established that these aberrant processes can result in dys- and hypo-myelination, our data are the first to demonstrate the dramatic effects on myo-inositol solute carriers which also may be responsible for myelin dysfunction.

## List of abbreviations

16:0: palmitic acid; 18:0: stearic acid; 18:1: oleic acid; 18:2: linoleic acid; 20:4: arachidonic acid; 22:6: docosahexaenoic acid (DHA); ER: endoplasmic reticulum; PLP: proteolipid protein; PlsEtn: ethanolamine plasmalogen; PMD: Pelizaeus-Merzbacher disease; SLC: solute carrier; SMIT: sodium-dependent myo-inositol transporter.

## Competing interests

The authors declare that they have no competing interests.

## Authors' contributions

All authors participated in the study design, supervision of assay QA/QC and data interpretation. TS and PW performed experiments. All authors read and approved the manuscript.
